# Bovine Rhinitis B Virus Variant as the Putative Cause of Bronchitis in Goat Kids

**DOI:** 10.3390/v16071023

**Published:** 2024-06-25

**Authors:** Andrew Noel, Jianqiang Zhang, Huigang Shen, Anugrah Saxena, Jennifer Groeltz-Thrush, Ganwu Li, Michael C. Rahe

**Affiliations:** 1 Veterinary Diagnostic and Production Animal Medicine, College of Veterinary Medicine, Iowa State University, Ames, IA 50011, USA; anoel@iastate.edu (A.N.);; 2 Department of Population Health and Pathobiology, College of Veterinary Medicine, North Carolina State University, Raleigh, NC 27607, USA

**Keywords:** bronchitis, picornavirus, caprine, bovine rhinitis B virus, zoonotic, cross-species transmission, genetic diversity

## Abstract

A diagnostic investigation into an outbreak of fatal respiratory disease among young goats in Iowa, USA revealed bronchitis lesions of unknown etiology and secondary bacterial bronchopneumonia. Hypothesis-free metagenomics identified a previously unreported picornavirus (USA/IA26017/2023), and further phylogenetic analysis classified USA/IA26017/2023 as an aphthovirus related to bovine rhinitis B virus. Viral nucleic acid was localized to lesions of bronchitis using in situ hybridization. This marks the first report of a picornavirus putatively causing respiratory disease in goats and highlights the potential for cross-species transmission of aphthoviruses.

## 1. Introduction

The family *Picornaviridae* includes a group of non-enveloped viruses, approximately 22 to 32 nm in virion diameter, with a single-stranded positive-sense RNA genome of 7–8.5 kb in length. The picornavirus genome is composed of one open reading frame encoding a polyprotein, a 5′ untranslated region involved in replication and translation processes, and a 3′ poly(A) tail. The polyprotein is processed into P1-3, with P1 containing structural elements and P2-3 associated with polyprotein processing and viral replication [1]. Members of this family are diverse in the host species, tissues, and cells that they infect as well as in the resulting clinical disease and pathology. Many picornaviruses are important human and veterinary pathogens, such as equine rhinitis A virus (ERAV, species *Aphthovirus burrowsi*), a member of the genus *Aphthovirus* [2,3]. This genus also includes foot-and-mouth disease virus (FMDV, species *Aphthovirus vesiculae*), bovine rhinitis A virus (BRAV, species *Aphthovirus bogeli*), and five genotypes of bovine rhinitis B virus (BRBV, species *Aphthovirus reedi*) [4]. BRBV is prevalent in cases of bovine respiratory disease and can cause upper respiratory tract infections in calves [5,6]. Viruses known to cause respiratory disease in sheep and goats include parainfluenza virus-3, Peste des petits ruminants, ovine respiratory syncytial virus, and bluetongue virus [7]. Here, we describe the discovery of a novel BRBV variant in a young goat using next-generation sequencing, metagenomic analysis, and direct-detection methods to implicate the aphthovirus as the cause of bronchitis amidst a herd-level outbreak of fatal respiratory disease.

## 2. Materials and Methods

### 2.1. Case History and Sample Collection

A goat dairy in Northeast Iowa experienced a respiratory disease outbreak resulting in the rapid death of several 3-week-old kids recently introduced into the premises. Following the death of two goat kids, necropsies were performed by the attending veterinarian. In both kids, the lungs were noted to have gross pathology lesions suggestive of bacterial pneumonia. The heart and lungs were removed, and cranioventral lung sections from each animal were collected in one jar of 10% neutral-buffered formalin. These formalin-fixed lung sections and the remainder of the heart and lung tissues were submitted to the Iowa State University Veterinary Diagnostic Laboratory (Ames, IA, USA) for diagnostic evaluation.

### 2.2. Routine Diagnostics

Upon arrival, the samples were accessioned and examined per procedures for a routine diagnostic case. Formalin-fixed bronchi were carefully dissected away from the lungs for separate examination. Lung tissue and bronchi were processed by routine histologic methods, embedded in paraffin, placed onto glass slides, and stained with hematoxylin and eosin for microscopic examination. Routine bacterial culture of intact lung tissue and PCR for bovine parainfluenza virus-3 (BPIV-3) on lung homogenate were performed using established methods [8].

### 2.3. Whole-Genome Sequencing, Metagenomics, and Phylogenetic Analysis

Following a diagnosis of bronchitis and faced with the absence of common differentials for that lesion in goats, next-generation sequencing (NGS) based on the hypothesis-free metagenomics approach was employed on the lung homogenate [9]. Recovered sequences were analyzed with a BLAST search against the NCBI database (the Basic Local Alignment Search Tool, the National Center for Biotechnical Information; Bethesda, MD USA).

A near-full-length novel picornavirus genome sequence of 7369 nucleotides (USA/IA26017/2023) was identified and deposited in GenBank with accession number OR906128. The MAFFT alignment of the MegAlign Pro 17 program in the DNASTAR Lasergene 17 software was utilized to compare USA/IA26017/2023 with bovine rhinitis B virus (BRBV) SC1_LZ02 isolate (GenBank #MZ052127), 24 other BRBV complete-genome sequences, and aphthovirus RBV-JN022 (GenBank #OQ547742), which was recently identified in sheep in China. Phylogenetic trees were constructed based on the whole-genome sequences and the VP1 sequences using the neighbor-joining method in MEGA version 6 [10] with p-distance as the substitution model. Bootstrap analysis was carried out with 1000 replicates. Representative sequences of different BRBV genotypes were obtained from the website “https://www.picornaviridae.com/caphthovirinae/aphthovirus/brbv/brbv_seq.htm (accessed on 19 June 2024)”. The plot for sequence comparison was performed using SimPlot software v. 3.5.1.

### 2.4. Virus Isolation, PCR, and In Situ Hybridization

Virus isolation using the lung tissue homogenate was attempted on Madin-Darby bovine kidney cells (MDBK; ATCC CCL-22) and bovine turbinate cells (BT; ATCC CRL-1390).

Published BRBV PCR assays targeting the 3Dpol or 3′UTR (untranslated region) were attempted on the lung homogenate and cell culture supernatant [11,12]. Additionally, an in-house real-time RT-PCR (forward primer TGTCCTTTGCACGGCGT; reverse primer AAAGCYCCCACAAACGGAG; probe /56-FAM/CAGGAGAAGATAACCTCTGTGGCGGGTCT/36-TAMSp/) was developed from the BRBV variant sequence obtained via NGS and attempted on the same specimens.

An RNAscope™ in situ hybridization (ISH) red chromogenic assay with an RNA probe specific to USA/IA26017/2023 was created by Advanced Cell Diagnostics (ACD; Newark, CA, USA) and performed using established methods on slides prepared from blocks of formalin-fixed, paraffin-embedded lung and bronchus tissue with lesions of bronchitis [13].

## 3. Results

### 3.1. Routine Diagnostics

**Figure 1:**
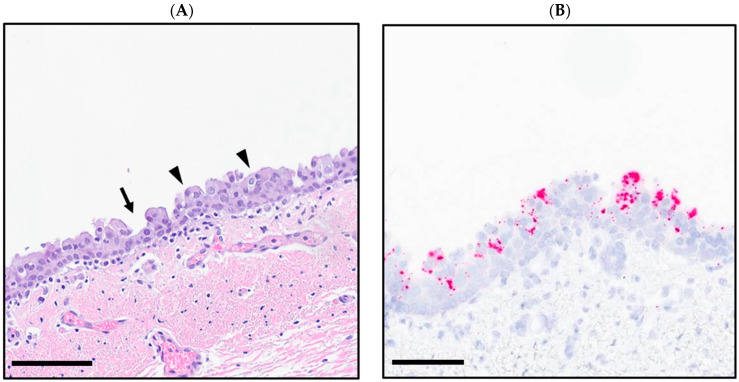

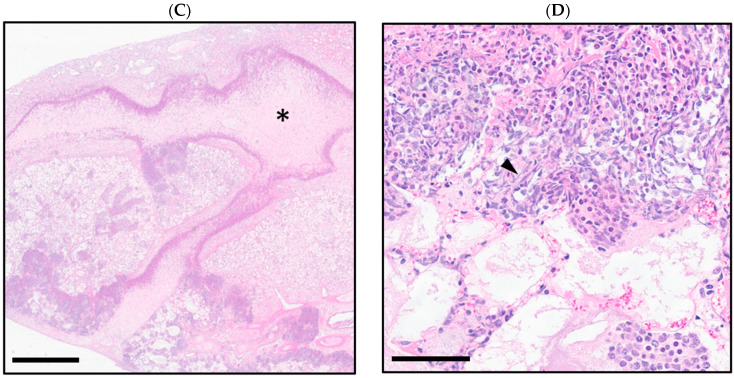
(**A**) BRBV variant-infected goat bronchial epithelium with antibasilar nuclei, loss of cilia (arrowheads), and segmental attenuation (arrow); (hematoxylin and eosin-stained; scale bar = 80 µm). (**B**) BRBV variant-infected goat bronchus; RNAscope in situ hybridization for BRBV variant (red; hematoxylin counterstain; scale bar = 60 µm). (**C**) Goat lung with multifocal, mosaic pattern of coagulative necrosis surrounded by degenerate leukocytes (asterisk) (hematoxylin and eosin-stained; scale bar = 1 mm). (**D**) Goat lung in (**C**), highlighting the presence of degenerate leukocytes with nuclear streaming (arrowhead) surrounding regions of necrosis (hematoxylin and eosin-stained; scale bar = 60 µm).

Grossly, the bilateral cranioventral regions of the lungs were dark purple, markedly consolidated, and in fair postmortem condition. Histopathologic examination of the bronchi revealed segments of hyperplastic and stacked respiratory epithelial cells up to three layers thick. Intact pseudostratified epithelial cells had antibasilar nuclei and loss of cilia. The subjacent lamina propria was infiltrated by medium numbers of lymphocytes and plasma cells (Figure 1A). Within the lung parenchyma, there were multifocal areas of necrosis and colonies of short rod-shaped bacteria surrounded by degenerate neutrophils and occasional oat cells (Figure 1C,D), consistent with infection by a leukotoxin-producing bacterial species. One such organism, *Mannheimia haemolytica*, was isolated at high growth in a routine culture of the affected tissue [14]. The PCR testing of the lung homogenate for BPIV-3 was negative.

### 3.2. Whole-Genome Sequencing, Metagenomics, and Phylogenetic Analysis

Within the lung, *Mannheimia haemolytica* (39,759 reads) and *Mycoplasma ovipneumoniae* (578 reads) were identified, along with a novel picornavirus, given the name USA/IA26017/2023 (608 reads). The assembled genome is 7402 nucleotides (nt) in length. It features a single large open reading frame (ORF) that encodes a polyprotein, subsequently cleaved into functional viral proteins. This ORF is divided into the leader protein (L), acting as a protease, the P1 region encoding structural proteins (VP1, VP2, VP3, and VP4) that form the viral capsid, and the P2 (2A-2B-2C) and P3 (3A-3B-3C-3D) regions, encoding non-structural proteins crucial for viral RNA replication and polyprotein processing. The layout of the detected USA/IA26017/2023 strain is as follows: 5‘ UTR—Leader (599–1219 nt)–VP4 (1220–1495 nt)–VP2 (1496–2179 nt)–VP3 (2180–2836 nt)–VP1 (2837–3493 nt)–2A (3494–3610 nt)–2B (3611–3991 nt)–2C (3992–4939 nt)–3A (4940–5338 nt)–3B (5339–5413 nt)–3C (5414–6037 nt)–3D (6038–7402 nt). The assembled sequence of USA/IA26017/2023 lacked the last 39 nucleotides in the coding region of the 3D protein.

As assembled, USA/IA26017/2023 exhibited 80.2% nucleotide identity with bovine rhinitis B virus (BRBV) SC1_LZ02 isolate (GenBank #MZ052127) and 72.2–78.4% nucleotide identity with 24 other BRBV complete-genome sequences as well as aphthovirus RBV-JN022. The remaining amino acid sequence of the polyprotein shared 90.3% identity with that of the BRBV SC1_LZ02 isolate. Furthermore, nucleotide and amino acid (aa) pairwise comparisons for different genome regions between the study strain USA/IA26017/2023, the most closely related bovine rhinitis B virus (BRBV) SC1_LZ02 isolate (MZ052127), and the reference strain EC11 (EU236594) were performed (Table S1). An aa distance plot was generated using SimPlot (Figure S1). At the aa level, the study strain USA/IA26017/2023 showed the highest identity in the 2C (96.5%) and 3B (96.0%) regions and the lowest identity in the 2A (73.7%) and VP1 (80.2%) regions compared with the SC1_LZ02 isolate (Table S1; Figure S1).

**Figure 2:**
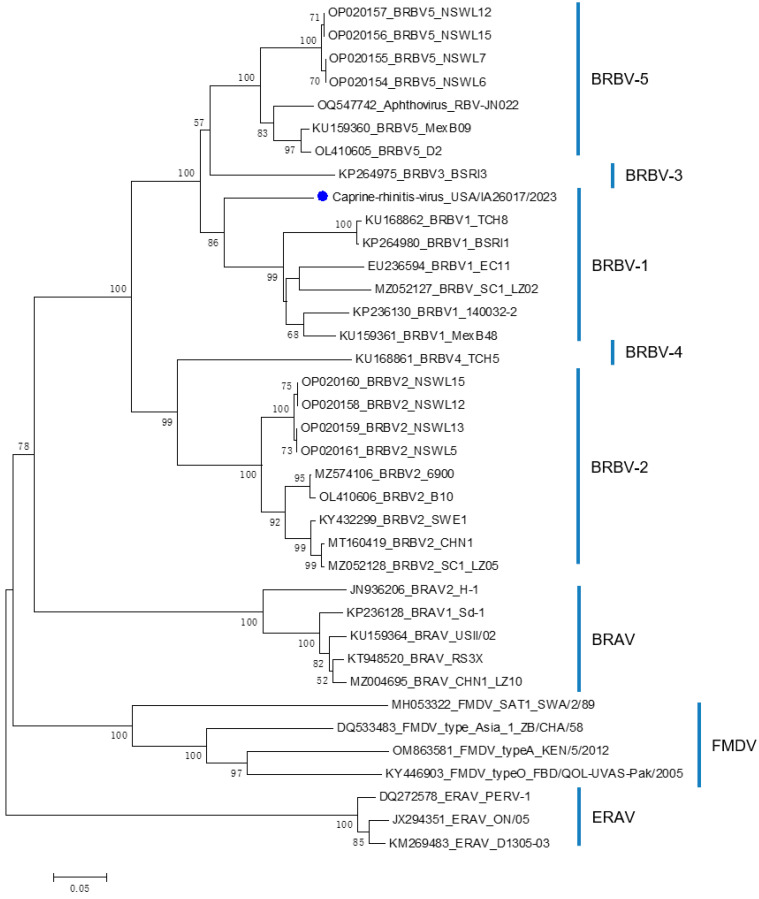
Phylogenetic analysis based on VP1 amino acid sequences. The phylogenetic tree was constructed by the neighbor-joining (NJ) method. Significant bootstrap values are indicated as percentages for 1000 replicates; bootstraps higher than 50 are displayed along the relative branches. BRAV: bovine rhinitis A virus; BRBV: bovine rhinitis B virus; ERAV: equine rhinitis A virus; FMDV: foot-and-mouth disease virus. The bovine rhinitis B virus variant USA/IA26017/2023 is indicated by a blue dot.

The construction of a phylogenetic tree based on the whole-genome sequences revealed USA/IA26017/2023 belongs to the genus *Aphthovirus* and is closely clustered with other BRBV sequences as well as the virus RBV-JN022, suggesting that the newly identified virus USA/IA26017/2023 is likely a BRBV variant (Figure S2). Furthermore, phylogenetic analysis based on the VP1 amino acid sequences suggested that USA/IA26017/2023 belongs to the BRBV-1 genotype (Figure 2). According to the official species demarcation criteria for aphthoviruses found at “https://ictv.global/report/chapter/picornaviridae/picornaviridae/aphthovirus (accessed 17 June 2024),” pairwise amino acid sequence identity analysis was performed on the complete polyprotein, P1, and 2C+3CD proteins. Compared with other BRBV isolates, USA/IA26017/2023 shows an identity of 83.5–90.3% for polyprotein, 70.5–88.5% for P1, and 91.3–94.7% for 2C+3CD. Specifically, USA/IA26017/2023 shows an identity of 90.3% for polyprotein, 88.5% for P1, and 92.4% for 2C+3CD compared with the SC1_LZ02 isolate (MZ052127). It also shows an identity of 89.6% for polyprotein, 86.5% for P1, and 93.1% for 2C+3CD compared with the reference strain EC11 (EU236594). Therefore, USA/IA26017/2023 exhibits less than 30% divergence in the polyprotein aa sequence, less than 40% divergence in the P1 aa sequence, and less than 20% divergence in the 2C+3CD aa sequence compared with the other BRBV reference strains, demonstrating that USA/IA26017/2023 is the same species as BRBV.

### 3.3. Virus Isolation, PCR, and In Situ Hybridization

Interestingly, published BRBV PCR assays failed to detect the BRBV variant in the lung homogenate from the goat specimen [11,12]. Using the in-house real-time RT-PCR specific for the BRBV variant sequence, the lung homogenate tested positive, with a Ct value of 26. When virus isolation was attempted in both MDBK and BT cell lines, no virus cytopathic effects were observed, and the cell culture supernatants after two passages were negative by BRBV variant PCR, suggesting that virus isolation attempts were unsuccessful.

Strong signals of red chromogenic RNAscope™ (ACD) ISH labeling for BRBV variant RNA were observed in the cytoplasm of stacked, degenerate, and adjacent bronchial epithelial cells (Figure 1B).

## 4. Discussion

In this goat case, the identification of a BRBV variant by next-generation sequencing within pneumonic tissue and the detection of viral RNA within microscopic lesions of bronchitis via ISH is evidence for this virus being the putative primary cause of clinical respiratory disease. Previous metagenomic research has associated BRBV with clinical bovine respiratory disease, and the tropism of the BRBV variant for the respiratory epithelium aligns with that of BRBV [5,15]. The epithelial regeneration in the bronchus suggests infection with the BRBV variant occurred days prior to death, before the acute necrotizing lesions of Mannheimiosis developed. Given that timeline, the BRBV variant infection of respiratory epithelial cells likely compromised the mucociliary apparatus, predisposing this animal to the ultimately fatal secondary bacterial pneumonia in a well-known interplay between viral and bacterial agents in ruminant respiratory disease [16]. Therefore, this BRBV variant should be considered as a differential diagnosis in caprine respiratory disease.

The differences in the NGS reads reported for the BRBV variant and *Mannheimia haemolytica* were due to several factors, including the pathogen distribution, sample type, and genome length. The BRBV variant ISH signal was limited to the bronchi, and a large proportion of terminal bronchioles and alveoli appeared to be damaged by the fulminant *M. haemolytica* infection, suggesting a high concentration of nucleic acids in those respective locations. Unfortunately, the only viable sample for NGS in this case was a fresh lung homogenate, which favored the detection of *M. haemolytica* over the BRBV variant. Furthermore, the BRBV variant lesions were chronic and resolving, while the Mannheimia lesions were acute and progressing. Given the genome of *M. haemolytica* is about 2.6 × 10^6^ bp, and the picornavirus genome is only about 7400 bp, approximately 350 times more *M. haemolytica* reads than BRBV variant reads are expected for each individual bacterial and viral unit present [17]. Considering the location, duration, and genome size, it is logical to conclude that there were abundant BRBV variant nucleic acids present in the lung homogenate.

A similar logic can be applied to the detection of *Mycoplasma ovipneumoniae*, which is widely distributed across sheep and goats and can be found in apparently healthy animals [18]. *M. ovipneumoniae* can be recovered from experimentally infected lambs without pathological changes, and clinical infection is typically chronic [19,20]. More reads of the BRBV variant were found than of *M. ovipneumoniae* despite its larger genome, and no lesions suggesting *M. ovipneumoniae* as a factor in the rapid death of this kid were observed [21].

To our knowledge, this report signifies the first documented case of a picornavirus localized to the respiratory tract of goats. Although it is theoretically possible that the BRBV variant found by ISH may have been replicating in lesions caused by another epitheliotropic virus, this is highly unlikely given the lack of other viral reads found in the metagenomic analysis. The origin of the BRBV variant remains unclear, as there was no indication that the affected goats had contact with cattle to serve as a reservoir for BRBV spillover. Interestingly, whole-genome phylogenetic analysis indicated that a virus, RBV-JN022, recently identified in sheep from China also clustered with the virus USA/IA26017/2023 and other BRBV sequences, suggesting that the virus RBV-JN022 is likely a BRBV variant as well. A novel picornavirus belonging to the same subfamily as BRBV was discovered in wild Australian fallow deer. This further demonstrates the wide host range of this family and the potential role of wildlife in the development of new viruses [22]. Additional studies are necessary to evaluate the prevalence and genetic diversity of these picornaviruses and their host species. Unfortunately, attempts to isolate the BRBV variant USA/IA26017/2023 in MDBK and BT cells were unsuccessful; isolation attempts in other cell lines are planned. Members of the *Aphthovirus* genus use various host receptors for viral entry including those in the immunoglobulin superfamily, α2,3-linked sialic acid, and integrin family [1,23,24]. FMDV, capable of infecting many ruminant species, has been shown to interact with members of the integrin family in vitro, and one of them, αvβ6, is constitutively expressed on epithelial cells in cattle [25,26,27]. The cell receptor for USA/IA26017/2023 remains unknown.

This study provides valuable insights into a potentially novel pathogenic picornavirus, warranting further investigations into its prevalence, distribution, and genetic diversity. Future steps include attempts to isolate this BRBV variant virus in susceptible cells, in vivo studies to fulfill Koch’s postulates, and the characterization of its pathogenesis. This discovery contributes to our understanding of picornaviruses, particularly the virus members of the genus *Aphthovirus.*

## Data Availability

The original contributions presented in this study are included in this article. Further inquiries can be directed to the corresponding authors.

## Supplementary Materials

The following supporting information can be downloaded at: https://www.mdpi.com/article/10.3390/v16071023/s1, Figure S1: Amino acid distance plot; Figure S2: Phylogenetic tree constructed with whole genome sequences; Table S1: Pairwise identities of BRBV variant strain USA/IA26017/2023 compared with other strains.

## References

[1] Tuthill T.J., Groppelli E., Hogle J.M., Rowlands D.J. (2010). Picornaviruses. Curr. Top. Microbiol. Immunol..

[2] Kriegshäuser G., Deutz A., Kuechler E., Skern T., Lussy H., Nowotny N. (2005). Prevalence of neutralizing antibodies to *Equine rhinitis* A and B virus in horses and man. Vet. Microbiol..

[3] Plummer G. (1963). An equine respiratory enterovirus. Some biological and physical properties. Arch. Gesamte Virusforsch..

[4] Lefkowitz E.J., Dempsey D.M., Hendrickson R.C., Orton R.J., Siddell S.G., Smith D.B. (2018). Virus taxonomy: The database of the International Committee on Taxonomy of Viruses (ICTV). Nucleic Acids Res..

[5] Bhattarai S., Lin C.M., Temeeyasen G., Palinski R., Li F., Kaushik R.S., Hause B.M. (2022). Bovine rhinitis B virus is highly prevalent in acute bovine respiratory disease and causes upper respiratory tract infection in calves. J. Gen. Virol..

[6] Hollister J.R., Vagnozzi A., Knowles N.J., Rieder E. (2008). Molecular and phylogenetic analyses of bovine rhinovirus type 2 shows it is closely related to foot-and-mouth disease virus. Virology.

[7] Li W., Mao L., Cheng S., Wang Q., Huang J., Deng J., Wang Z., Zhang W., Yang L., Hao F. (2014). A novel parainfluenza virus type 3 (PIV3) identified from goat herds with respiratory diseases in eastern China. Vet. Microbiol..

[8] Horwood P.F., Mahony T.J. (2011). Multiplex real-time RT-PCR detection of three viruses associated with the bovine respiratory disease complex. J. Virol. Methods.

[9] Chen Q., Wang L., Zheng Y., Zhang J., Guo B., Yoon K.J., Gauger P.C., Harmon K.M., Main R.G., Li G. (2018). Metagenomic analysis of the RNA fraction of the fecal virome indicates high diversity in pigs infected by porcine endemic diarrhea virus in the United States. Virol. J..

[10] Tamura K., Stecher G., Peterson D., Filipski A., Kumar S. (2013). MEGA6: Molecular Evolutionary Genetics Analysis version 6.0. Mol. Biol. Evol..

[11] Kishimoto M., Tsuchiaka S., Rahpaya S.S., Hasebe A., Otsu K., Sugimura S., Kobayashi S., Komatsu N., Nagai M., Omatsu T. (2017). Development of a one-run real-time PCR detection system for pathogens associated with bovine respiratory disease complex. J. Vet. Med. Sci..

[12] Zhou Y., Chen X., Tang C., Yue H. (2023). Detection and Genomic Characterization of Bovine Rhinitis Virus in China. Animals.

[13] Rahe M.C., Magstadt D.R., Groeltz-Thrush J., Gauger P.C., Zhang J., Schwartz K.J., Siepker C.L. (2022). Bovine coronavirus in the lower respiratory tract of cattle with respiratory disease. J. Vet. Diagn. Investig..

[14] Ackermann M.R., Brogden K.A. (2000). Response of the ruminant respiratory tract to *Mannheimia* (*Pasteurella*) *haemolytica*. Microbes Infect..

[15] Zhang M., Hill J.E., Fernando C., Alexander T.W., Timsit E., van der Meer F., Huang Y. (2019). Respiratory viruses identified in western Canadian beef cattle by metagenomic sequencing and their association with bovine respiratory disease. Transbound. Emerg. Dis..

[16] Mosier D. (2014). Review of BRD pathogenesis: The old and the new. Anim. Health Res. Rev..

[17] Gioia J., Qin X., Jiang H., Clinkenbeard K., Lo R., Liu Y., Fox G.E., Yerrapragada S., McLeod M.P., McNeill T.Z. (2006). The genome sequence of *Mannheimia haemolytica* A1: Insights into virulence, natural competence, and Pasteurellaceae phylogeny. J. Bacteriol..

[18] Maksimović Z., Rifatbegović M., Loria G.R., Nicholas R.A.J. (2022). Mycoplasma ovipneumoniae: A Most Variable Pathogen. Pathogens.

[19] Johnson T., Jones K., Jacobson B.T., Schearer J., Adams N., Thornton I., Mosdal C., Jones S., Jutila M., Rynda-Apple A. (2022). Experimental infection of specific-pathogen-free domestic lambs with Mycoplasma ovipneumoniae causes asymptomatic colonization of the upper airways that is resistant to antibiotic treatment. Vet. Microbiol..

[20] DaMassa A.J., Wakenell P.S., Brooks D.L. (1992). Mycoplasmas of goats and sheep. J. Vet. Diagn. Investig..

[21] Yang F., Tang C., Wang Y., Zhang H., Yue H. (2011). Genome sequence of Mycoplasma ovipneumoniae strain SC01. J. Bacteriol..

[22] Huaman J.L., Pacioni C., Sarker S., Doyle M., Forsyth D.M., Pople A., Carvalho T.G., Helbig K.J. (2021). Novel Picornavirus Detected in Wild Deer: Identification, Genomic Characterisation, and Prevalence in Australia. Viruses.

[23] Neff S., Sá-Carvalho D., Rieder E., Mason P.W., Blystone S.D., Brown E.J., Baxt B. (1998). Foot-and-mouth disease virus virulent for cattle utilizes the integrin alpha(v)beta3 as its receptor. J. Virol..

[24] Fry E.E., Tuthill T.J., Harlos K., Walter T.S., Rowlands D.J., Stuart D.I. (2010). Crystal structure of *Equine rhinitis* A virus in complex with its sialic acid receptor. J. Gen. Virol..

[25] Jackson T., Sheppard D., Denyer M., Blakemore W., King A.M. (2000). The epithelial integrin alphavbeta6 is a receptor for foot-and-mouth disease virus. J. Virol..

[26] Duque H., LaRocco M., Golde W.T., Baxt B. (2004). Interactions of foot-and-mouth disease virus with soluble bovine alphaVbeta3 and alphaVbeta6 integrins. J. Virol..

[27] Monaghan P., Gold S., Simpson J., Zhang Z., Weinreb P.H., Violette S.M., Alexandersen S., Jackson T. (2005). The alpha(v)beta6 integrin receptor for Foot-and-mouth disease virus is expressed constitutively on the epithelial cells targeted in cattle. J. Gen. Virol..

