# Supplementary material for: Bovine Rhinitis B Virus Variant as the Putative Cause of Bronchitis in Goat Kids
Source: Viruses. 2024 Jun 25;16(7):1023. doi: 10.3390/v16071023 (PMC11281505; doi:10.3390/v16071023)
Supplement: Supplementary file 1 [file viruses-16-01023-s001.zip › viruses-2997442-supplementary.pdf]

Figure S1. Amino acid distance plot

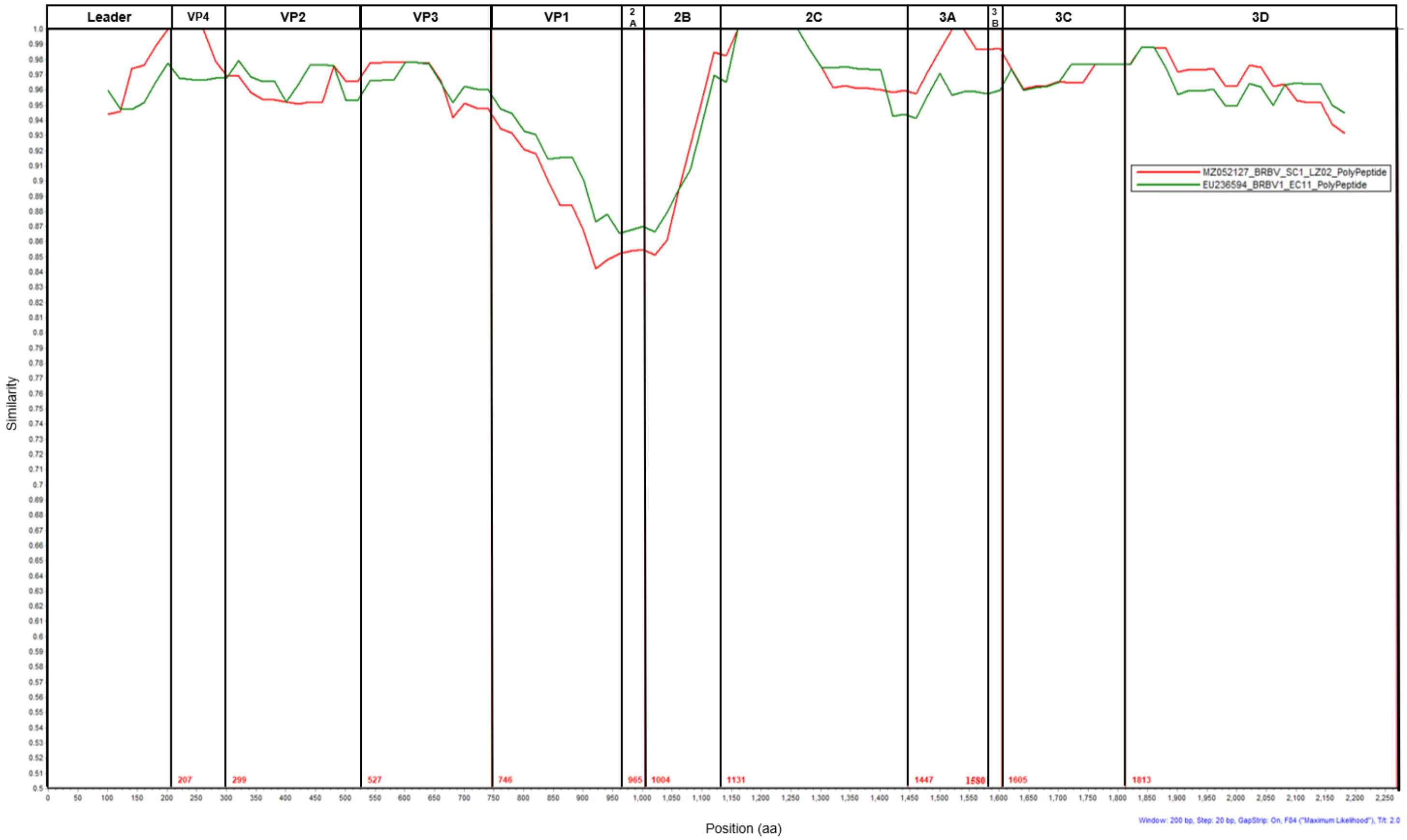

Figure S1. Amino acid distance plot comparing the BRBV SC1_LZ02 isolate (MZ052127, red solid line) and the reference strain EC11 (EU236594, green solid line) to the BRBV variant strain USA/IA26017/2023 used as the query sequence. The SimPlot analysis covers the amino acid sequences of the polyprotein from the "Leader" to "3D" proteins. Black solid lines indicate the amino acid positions within the polyprotein.

Figure S2. Phylogenetic tree constructed with whole genome sequences

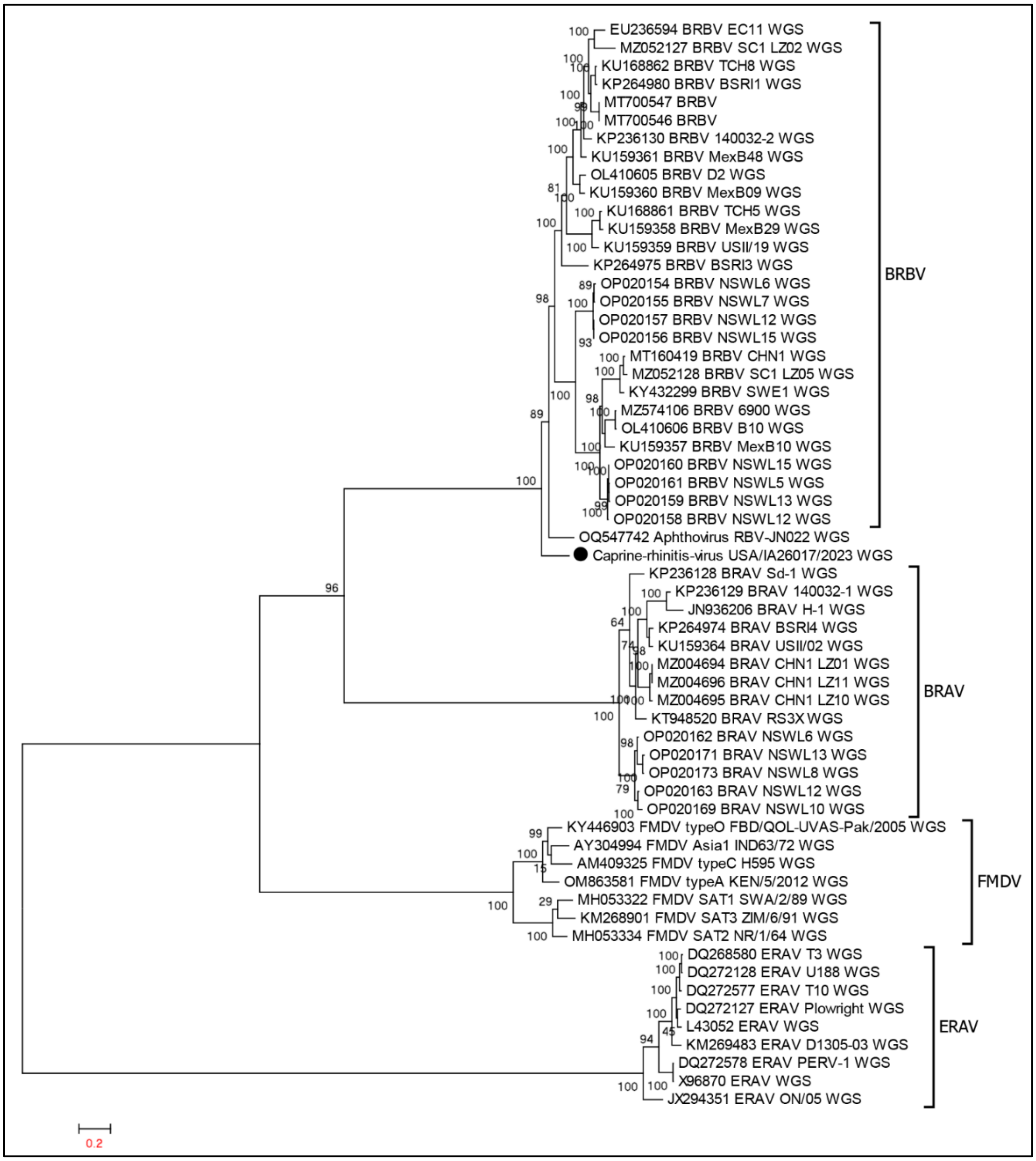

Figure S2. Phylogenetic tree showing the bovine rhinitis B virus variant (bullet point) and other aphthovirus species based on whole-genome sequences. Constructed by the maximum likelihood model and evaluated fitness by bootstrapping with 1000 replicates using IQTree-2.2.2.6 and then annotated in MEGA version 6 [8]. BRAV: bovine rhinitis A virus; BRBV: bovine rhinitis B virus; ERAV: equine rhinitis A virus; FMDV: foot-and-mouth disease virus.

Table S1. Pairwise identities of BRBV variant strain USA/IA26017/2023 compared with other strains

Genome	nt Identity		aa Identity	
Regions	MZ052127 (SC1_LZ02)	EU236594 (EC11)	MZ052127 (SC1_LZ02)	EU236594 (EC11)
Polyprotein	79.7	79.2	90.3	89.6
Leader	77.7	78.9	87.4	87.0
VP4	84.4	81.5	94.6	94.6
VP2	80.3	76.8	89.5	86.4
VP3	80.1	78.7	94.1	92.7
VP1	73.8	72.7	80.2	77.5
2A	73.7	74.8	73.7	76.3
2B	81.3	80.8	94.5	91.3
2C	82.6	82.5	96.5	97.5
3A	82.3	79.6	90.2	88.7
3B	78.7	84.0	96.0	92.0
3C	80.9	80.1	90.9	93.3
3D	80.7	81.3	90.3	89.9

Legend: nt, nucleotides; aa, amino acid sequence.
